# Enhancing the Hypolipidemic and Functional Properties of *Flammulina velutipes* Root Dietary Fiber via Steam Explosion

**DOI:** 10.3390/foods13223621

**Published:** 2024-11-13

**Authors:** Chao Ma, Liying Ni, Mengxue Sun, Fuxia Hu, Zebin Guo, Hongliang Zeng, Wenlong Sun, Ming Zhang, Maoyu Wu, Baodong Zheng

**Affiliations:** 1 College of Food Science, Fujian Agriculture and Forestry University, Fuzhou 350002, China; mch.01@163.com (C.M.); gzb8607@163.com (Z.G.); zhlfst@163.com (H.Z.); 2 Jinan Fruit Research Institute, All-China Federation of Supply and Marketing Co-Operatives, Jinan 250014, China; niliying12@163.com (L.N.); smx18313941107@163.com (M.S.); h18864801380@163.com (F.H.); zhangming_101@126.com (M.Z.); 3 School of Life Sciences and Medicine, Shandong University of Technology, Zibo 255000, China; wenlong3420@126.com

**Keywords:** *Flammulina velutipes* root, dietary fiber, physicochemical properties, obese mice

## Abstract

*Flammulina velutipes* is an edible mushroom widely cultivated in China. As a by-product of *Flammulina velutipes*, the roots are rich in high-quality dietary fiber (DF). In order to obtain high-quality soluble dietary fiber (SDF), steam explosion (SE) is used as an effective modification method to improve the extraction rate and avoid the loss of active substances. Mounting evidence shows that SDF alleviates lipid metabolism disorders. However, it is not well understood how the influence of SDF with SE pretreatment could benefit lipid metabolism. In this study, we extracted a soluble dietary fiber from *Flammulina velutipes* root with an SE treatment, named SE-SDF, using enzymatic assisted extraction. The physicochemical and structural properties of the SE-SDF were investigated, and its hypolipidemic effects were also analyzed using oleic-acid-induced HepG2 cells. In addition, the anti-obesity and hypolipidemic effects of SE-SDF were investigated using a high-fat diet (HFD) mouse model. The results indicate that SE treatment (1.0 MPa, 105 s) increased the SDF content to 8.73 ± 0.23%. The SE-SDF was primarily composed of glucose, galactose, and mannose. In HFD-fed mice, SE-SDF significantly reduced weight gain and improved lipid profiles, while restoring liver function and reducing injury. This work provides an effective method for the processing of fungi waste and adds to its economic value. In future studies, the structural characteristics and the anti-obesity and gut microbiota regulation mechanisms of SE-SDF will be explored in depth, supporting its high-value utilization in healthcare products.

## 1. Introduction

Obesity has a high incidence and has become a global health problem, posing a serious challenge to the world’s healthcare system. The World Obesity Atlas 2023 predicted that the number of obese and overweight people over 5 years old will reach 1.91 billion worldwide, and 18% of all adults in China will be obese by 2035 [1,2]. It is noteworthy that obesity is a key risk factor for metabolic diseases like hyperlipidemia, diabetes, atherosclerosis, cardiovascular disease, non-alcoholic fatty liver disease, and various other chronic diseases. The common methods for treating obesity and hyperlipidemia mostly involve surgery and pharmacological treatment. The main medications with which to control obesity are the appetite suppressants sibutramine and semaglutide and the pancreatic lipase inhibitor orlistat [3,4]. However, these drugs often cause adverse reactions involving vomiting, nausea, and gastrointestinal discomfort, which make long-term treatment difficult. Surgical interventions such as gastric ligation, liposuction, or intragastric hydrosphere bariatric surgery have shown potential benefits in obesity control. Nevertheless, surgical interventions bring risks of infection, bleeding, malnutrition, and anemia [5]. Therefore, finding an effective and safe method to treat obesity is urgent.

Increasing evidence demonstrates that the increased intake of dietary fiber (DF) in the daily diet could have a positive effect on diabetes prevention and weight control [6]. DF is divided into two types: soluble dietary fiber (SDF) and insoluble dietary fiber (IDF). Previous research reported that SDF mainly refers to the storage of substances and secretions in plant cells. It not only plays an important role in maintaining dietary balance but also participates in metabolic reactions in the human body and has important functional effects on preventing colon cancer and cardiovascular disease, as well as lowering cholesterol and blood lipid levels. However, IDF mainly plays a physical role in promoting gastrointestinal motility [7]. Furthermore, IDF may have some outstanding problems, such as rough taste, poor water-holding capacity, and low physiological activity, which have seriously limited the development and utilization of dietary fiber resources. Liu et al. [8] found that SDF, IDF, and TDF from bergamot effectively improved the dyslipidemia caused by high-fat diets in rats. Among them, the effect of the SDF group at the same dose is the best. The above studies revealed that SDF has a more significant effect on human health than IDF. However, the content of SDF is only around 3–4% of the total DF. Thus, finding a way to transform IDF into SDF is urgent.

*Flammulina velutipes* is an edible and medicinal fungus widely cultivated in China [9]. Previous studies have demonstrated that *F. velutipes* has rich nutritional value and biological properties, such as immunomodulatory [10,11], antitumor [12], and antioxidant activities [13]. Currently, large-scale artificial factory cultivation of *F. velutipes* has been established in Asia. The increasing cultivation of *F. velutipes* results in the overproduction of *F. velutipes* root, which is the main waste after the harvesting of the edible fruiting bodies of the fungi. A small proportion of this is processed into animal feed, but most is discarded. This practice is not only wasteful and harmful to the environment but also contravenes contemporary green and sustainable food production. Polysaccharide is a major active ingredient in *F. velutipes* root, which is divided into DF and active polysaccharides. Research on the potential health benefits of DF in disease prevention has received significant attention, and DF has been shown to have potential efficacy in alleviating obesity, type II diabetes, and cardiovascular diseases [14].

Steam explosion (SE) is a thermochemical–physical treatment using water as the transfer and reaction medium to enable the hydrolysis of cell wall components and the disruption of the cell wall structure in plant and fungi materials. Both the steaming and explosion stages lead to the compulsive and abundant release of natural products. In comparison with conventional procedures, like enzymatic and acid-based treatments, steam explosion is more efficient in terms of improving the extraction rate and avoiding the loss of active substances. Previous studies have reported that SE leads to the conversion of IDF to SDF and increases the extraction yield and efficiency of SDF originating from wheat bran and apple pomace owing to the hydrothermal deconstruction and conversion effects of SE [15,16]. SE treatment has been reported to reduce the molecular weight of *Ampelopsis grossedentata* polysaccharides, implying that some high-molecular-weight polysaccharides could be degraded into low-molecular-weight polysaccharides and oligosaccharides, leading to an increase in SDF [17]. Sui et al. [18] reported that the SE process caused the removal of hemicelluloses, the degradation of the cellulosic amorphous region, and the enhanced thermal stability of broccoli wastes.

Regarding the extraction of dietary fibers from fungi, methods such as enzymatic hydrolysis and acid–alkaline extraction have previously been explored. Enzymatic extraction, which uses specific enzymes to degrade the cell wall components and release fibers, can offer higher yields and specificity. Previous studies have shown that the fibers extracted from *F. velutipes*, using the enzymatic method, can increase the polysaccharide content and its hypolipidemic properties [19]. However, this method often requires expensive enzymes and precise control of conditions like temperature, pH, and reaction time, which limits the scalability of enzymatic hydrolysis in industrial applications.

Berktas et al. [20] evaluated the SDF yield via the acid and alkaline extraction method from quince. Their results showed that the yield was 4.18% (acid extraction) and 5.61% (alkali extraction), respectively. The purity of alkali-extracted SDF was decreased, which is most likely due to the high amount of ash in the final product coming from NaOH. Smiderle et al. [21] studied the alkaline extraction of polysaccharides from *F. velutipes* and found that while the method significantly increased the yield of hemicellulose and other fibers, it also resulted in a marked reduction in antioxidant activity due to the breakdown of β-glucans. Another study found that the acid–alkaline extraction significantly increased the extraction rate of polysaccharides from *Ganoderma lucidum* but reduced the structural integrity of the polysaccharides, which led to a decrease in their antioxidant and anti-inflammatory activities [22]. Although the high efficiency of SE-assisted extraction for natural products from plant materials has been confirmed, its effectiveness on edible mushrooms and the subsequent changes in their nutritional function need to be further investigated.

As a by-product of *F. velutipes*, the root is rich in DF and could be utilized to prepare high-quality SDF. In this work, we investigated the chemical properties and structural characteristics of SDF extracted from *F. velutipes* with an SE pretreatment (named SE-SDF). The potential anti-hyperlipidemia effect of the SE-SDF in vitro was primarily measured on oleic acid-induced HepG2 cells. In addition, physicobiochemical indicators and tissue morphology were evaluated in obese mice fed SE-SDF. Our results provide a scientific basis for the use of *F. velutipes* root SDF as a functional food ingredient and suggest an alternative strategy by which to alleviate hyperlipidemia.

## 2. Materials and Methods

### 2.1. Materials and Reagents

The *F. velutipes* root was purchased from Shandongyouhe Co., Ltd. (Jining, China). The 3-(4,5)-dimethylthiazol-2-yl)-2,5-diphenyltetrazolium bromide (MTT), Griess reagents, dimethyl sulfoxide (DMSO), and monosaccharide standard were purchased from Sigma Chemical Co., Ltd. (Shanghai, China). Dulbecco’s modified Eagle’s medium (DMEM) and fetal bovine serum (FBS) were obtained from Gibco (Grand Island, NY, USA). Thermostable a-amylase (A8751, 40,000 U/g), neutral protease (Z8031, 50,000 U/g), amyloglucosidase (T8500, 100,000 U/g), penicillin–streptomycin (P/S), and phosphate-buffered saline (PBS) were obtained from Solarbio Co., Ltd. (Beijing, China). The BCA protein assay kit and the detection kits for TC, TG, HDL-C, LDL-C, ALT, AST, GSH, SOD, CAT, and MDA were purchased from Nanjing Jiancheng Co., Ltd. (Nanjing, China). ELISA kits for LPS and IL-6 were obtained from Cusabio Co., Ltd. (Wuhan, China). The mouse feed was purchased from Synergy Pharmaceuticals Bioengineering Co., Ltd. (Nanjing, China). All the other reagents were analytical grade.

### 2.2. Steam Explosion Modification Process

**Figure 1:**
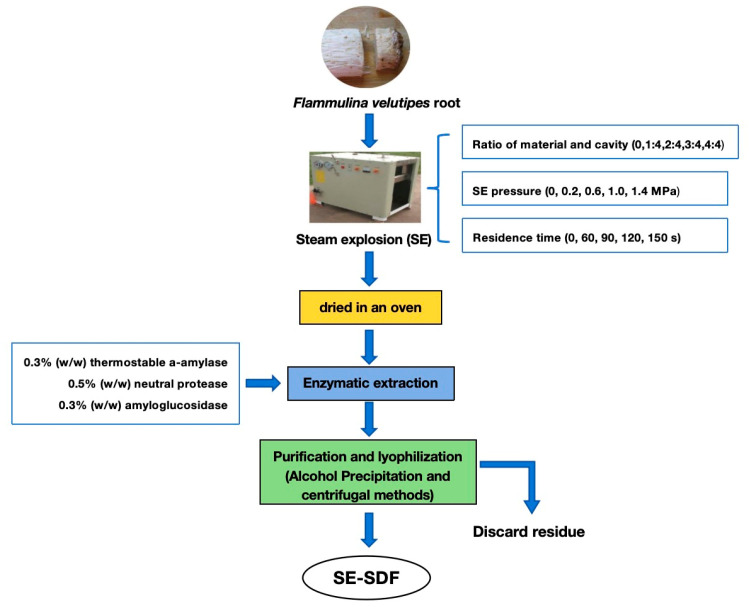
Steam explosion (SE) and extraction flowchart for SDF from *F. velutipes* root.

Pre-experiments used SDF yield as an indicator to explore material-to-cavity ratios (1:4 to 4:4), SE pressures (0.2 to 1.4 MPa), and residence times (60 to 150 s) on the SE modification of *F. velutipes* root SDF. The specific process optimization data are shown in Figure S1 and Table S1. According to the results, the optimized conditions were the ratio of material and cavity: 5:8; SE pressure: 1.0 MPa; and residence time 105 s. After SE pretreatment, the *F. velutipes* root was collected and oven-dried at 60 °C and stored at −20 °C. The SE and extraction flowchart of SDF from the *F. velutipes* root is illustrated in Figure 1.

### 2.3. SDF Extraction

The *F. velutipes* root, with or without SE pretreatment, was ground into powder and sifted through a 60 mesh. The extraction of SDF was conducted according to the enzymatic method of AOAC 991.43 [23], with slight modifications. Thermostable a-amylase, neutral protease, and amyloglucosidase were added successively to break down the protein and starch in the raw materials. The *F. velutipes* root powder, dispersed in a 30 times volume of ultrapure water, had 0.3% (*w*/*w*) thermostable a-amylase added at 95 °C, pH 6.0, under constant stirring of 120 rpm for 60 min. Then, the sample was enzymatically digested with 0.5% (*w*/*w*) neutral protease at 50 °C, pH 6.5, under constant stirring of 120 rpm for 60 min. After the temperature increased to 60 °C, 0.3% (*w*/*w*), amyloglucosidase was added and underwent further hydrolysis for 60 min at pH 6.5 under constant stirring of 120 rpm. Finally, the hydrolysate was extracted at 95 °C for 120 min, clarified via centrifugation at 4000 rpm for 10 min, and filtered with Whatman filter paper to remove any residue. The resulting supernatant was concentrated using a rotary evaporator. Then, the concentrated supernatant was mixed with 95% (*v*/*v*) ethanol at 4 °C for 12 h and centrifuged at 4000 rpm for 5 min. The precipitated flocculate was lyophilized to obtain SDF. The SDF extracted from *F. velutipes* root powder after SE was labeled SE-SDF.

The carbohydrate, protein, and phenol contents of SDF and SE-SDF were determined [24,25,26].

### 2.4. Structure Analysis

#### 2.4.1. Monosaccharide Composition Determination

The monosaccharide composition was analyzed according to Zhang et al. [27], with some modifications. Prior to analysis in a 6890 N gas chromatographic system (Agilent 6890 N, Agilent, Santa Clara, CA, USA), the SDF and SE-SDF were completely hydrolyzed and converted into acetylated aldononitrile derivatives. The monosaccharide standards (Fuc, Man, Glc, Gal, Xyl, Ara, and Rha) were also successively converted into acetylated aldononitrile derivatives using acetic anhydride–pyridine at 100 °C for 1 h. The following program was adopted for GC analysis: injection temperature: 250 °C; detector temperature: 280 °C. The velocities of N_2_, H_2_, and air were 1.2, 30, and 300 mL/min, respectively.

#### 2.4.2. Molecular Weight Analysis

Molecular weight analysis was performed with an Agilent 1260 InfinityⅡ MDS system (Agilent, USA) coupled with PL aquagel-OH Mixed-H 8 μm (300 mm × 7.5 mm, Agilent, USA) and a refractive index detector (DAWN HELEOS-II, Wyatt Technology Co., Santa Barbara, CA, USA). The sample was eluted by a 0.1 M NaNO_3_ aqueous solution at 45 °C. The flow rate was 1.0 mL/min [28].

#### 2.4.3. Infrared (IR) Spectroscopic Analysis

The SDF or SE-SDF was mixed with KBr, ground, and pressed into 1 mm pellets for analysis. The IR spectra were scanned in the range of 400 to 4000 cm^−1^ on a Nicolet 67 spectrometer (Thermo, Waltham, MA, USA).

#### 2.4.4. Scanning Electron Microscopy (SEM)

The SDF and SE-SDF samples were glued to a conductive adhesive, which was fixed on the sample stage. The samples were then gold-coated [29]. The SEM images were observed using a ZeissSupra55 scanning electron microscope (Shanghai Xiangyi Precision Instrument Co., Ltd., Shanghai, China).

### 2.5. Determination of Hyperlipidemic Effects of SDF and SE-SDF on HepG2 Cells

#### 2.5.1. Cell Culture and Cell Viability Analysis

HepG2 cells (CL-0103; Procell, Wuhan, China) were cultured in DMEM supplemented with 10% FBS and 1% P/S at 37 °C in a humidified incubator (HERAcell 150i, Thermo, USA) with 5% CO_2_. The effects of SDF or SE-SDF on cell viability were determined via MTT assay. Briefly, HepG2 cells were, respectively, seeded into 96-well plates at a density of 5 × 10^4^ cells/well. After incubation for 24 h, the cells were treated with SDF or SE-SDF at final concentrations of 0, 200, 400, or 800 μg/mL for another 24 h. All SDF or SE-SDF was dissolved in DMEM. At the end of the culture, 2 mg/mL of MTT was added to the cells for 4 h at 37 °C. Then, the absorbance was determined by an ELISA plate reader (BioTek PowerWave XS, Winooski, VT, USA) at 490 nm [30]. The experiment was performed three times.

#### 2.5.2. Determination of Intracellular Triglyceride (TG) and Cholesterol (TC) Content

HepG2 cells were seeded into six-well plates at a density of 1 × 10^6^ cells/well for 24 h, treated with various concentrations (0, 200, 400, or 800 μg/mL) of SDF or SE-SDF, and stimulated with 1 mmol/L of oleic acid (OA) for 24 h at 37 °C in a CO_2_ incubator. The total protein and TG and TC contents in the supernatant were measured with a BCA assay and TG and TC test kits, respectively. The experiment was performed three times.

### 2.6. Experimental Design for the Mouse Obesity Model

Six-week-old male C57BL/6J mice were provided by Ji’nan Pengyue Laboratory Animal Breeding Co., Ltd. (Jinan, China). All the mice were housed under conditions of 22 ± 2 °C, 50–60% humidity, and a 12/12 h light/dark cycle and had free access to water and food. After 1 week of adaptation, mice were randomly allocated into 5 groups (8 mice per group): ND group, fed the normal diet (19.2% protein, 67.3% carbohydrate, and 4.3% fat for a total calorie ratio of 3.85 kcal/gm) and 0.2 mL saline; HFD group, fed a high-fat diet (comprising 26% protein (calorie percentage 20 kcal%), 35% fat (calorie percentage 60 kcal%), and 26% carbohydrates (calorie percentage 20 kcal%) for a total calorie ratio of 5.24 kcal/gm) and 0.2 mL saline; SE-SDFH group, administered the HFD and 0.2 mL SE-SDF solution (200 mg/kg/day SE-SDF); SE-SDFM group, administered the HFD and 0.2 mL SE-SDF solution (100 mg/kg/day SE-SDF); and SE-SDFL group, administered the HFD and 0.2 mL SE-SDF solution (50 mg/kg/day SE-SDF). The specific compositions of the normal diet and high-fat diet are listed in Table S2. All the mice were treated via oral administration for 10 weeks.

**Figure 2:**
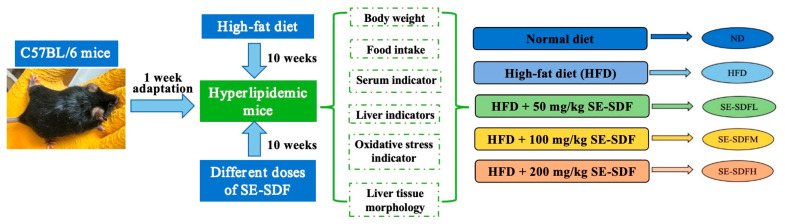
The animal experimental design of the study.

After the experiment started, the body weights of mice were recorded weekly. The average daily food intake of mice was recorded in the last week. At the end, all mice were fasted and had free access to water for 16 h. After weighing, they were euthanized with CO_2_. The blood was collected and centrifuged at 15,000 rpm for 5 min at 4 °C to obtain serum, which was stored at −80 °C for a later experiment. The body and liver weights of the experimental mice were recorded to calculate their liver index, according to Yue et al. [31]. In addition, part of the liver was fixed with 4% paraformaldehyde, and the other part was frozen in liquid nitrogen and stored at −80 °C. The animal experimental design of the study is shown in Figure 2.

### 2.7. Biochemical Analysis

The levels of total triglycerides (TG), total cholesterol (TC), low-density lipoprotein cholesterol (LDL-C), and high-density lipoprotein cholesterol (HDL-C) in the serum and liver were determined via commercially available kits. The levels of the liver function indicators alanine aminotransferase (ALT) and alanine transaminase (AST) in the serum were obtained through the kits. The level of hepatic oxidative stress was evaluated by assaying superoxide dismutase (SOD) and catalase (CAT) and glutathione (GSH), and malondialdehyde (MDA) in mice livers according to the instructions of each kit. The concentrations of inflammatory cytokines (lipopolysaccharide (LPS) and interleukin 6 (IL-6)) were determined by ELISA kits.

### 2.8. Histopathology Analysis of Liver

HE staining and Oil Red O staining assays of the liver were undertaken, according to Zheng et al. [32]. The images were captured by a microscope. The vacuolation and lipid accumulation degree were quantified using the Image J 1.53 program.

### 2.9. Statistical Analysis

All the experiments were performed in triplicate. The data were analyzed using SPSS 17.0. The results are expressed as mean ± SD and analyzed via one-way ANOVA and Tukey’s multiple comparison test with 95% confidence intervals. *p* < 0.05 was regarded as significant.

## 3. Results and Discussion

### 3.1. Physicochemical Properties of SDF Extracted from F. velutipes

The SDF from *F. velutipes* was extracted via the enzymatic method with a yield of 4.64 ± 0.11% based on dried weight. After SE pretreatment, the SE-SDF was collected with a yield of 8.73 ± 0.23%, representing an increase of 88.15% over that of the untreated sample. The chemical content, monosaccharide composition, and molecular weight of SDF and SE-SDF were determined (Table 1). The SE treatment increased the carbohydrate, phenol, and protein contents from 82.68% to 87.71%, 0.68% to 0.84%, and 2.16% to 3.51%, respectively. SE can break the barrier structure of the plant cell wall, prompting the solubilization and hydrolysis reactions of the ingredients [33]. The increment of phenols may be due to the disruption and hydrolysis of the ester bonds between the phenol and polysaccharides in the cell wall, resulting in the release of bound phenolics, while the increment of protein content may be due to the SE promoting the release of polysaccharides that are partially tightly linked to proteins [34]. Previous studies reported that phenolic and protein compounds possess hypolipidemic and anti-inflammatory activities, correlating with their impacts on enzymatic and signaling systems and their actions as antioxidants [35,36,37]. Song et al. [38] reported a similar hypolipidemic action of phenolic compounds and verified that the phenolic compounds extracted from Shanxi-aged vinegar alleviated hypolipidemic activities by regulating the PPARα-LXRα-ABCA1 pathway. Therefore, the change in SDF composition may enhance its hypolipidemic activity.

In addition, the monosaccharide composition results demonstrated that SDF and SE-SDF were heteroglycans, mainly consisting of glucose, galactose, and mannose. The molar ratio percentage of xylose and mannose after SE was 14.66% and 17.91%, which was 1.58 and 2.03 times that of the sample without SE pretreatment. This may be because the high-pressure and thermoacidic environment in the SE process disrupted the cellulose and hemicellulose structures, caused massive hydrolysis of the hemicelluloses, and thereby promoted xylose and mannose dissolution [15]. Previous studies reported that xylose and mannose exhibit various pharmacological activities, especially a hypolipidemic effect [39,40]. For example, the xylose and mannose from *Rosae laevigatae* fructus reduced the levels of TC, TG, and LDL-C while increasing the antioxidant lipids and upregulating the expression of PPAR-γ and LPL in hyperlipidemia rats [41].

### 3.2. Fourier Transform IR Analysis

**Figure 3:**
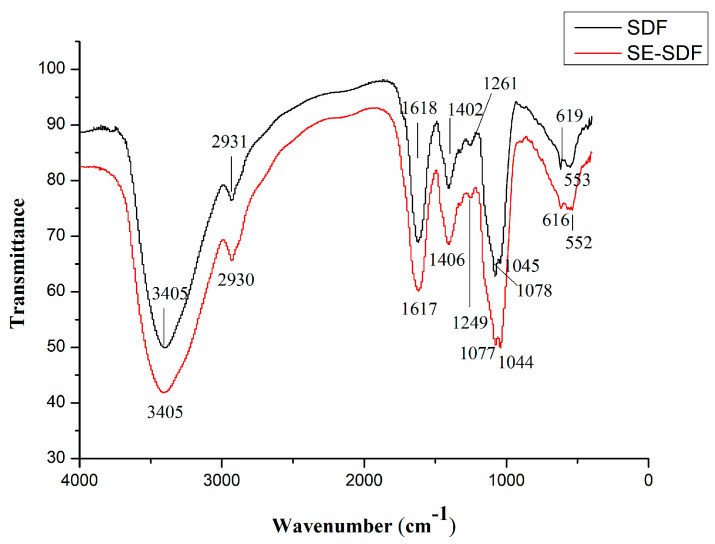
Characterization of the structural features of SDF and SE-SDF using FTIR.

As depicted in Figure 3, a broad, intense peak at 3405 cm^−1^ corresponded to the stretching vibration of the O–H linkage, indicating the strong inter- and intra-molecular interactions of polysaccharide chains and phenols [42]. The intensity of this peak is increased after SE treatment and may be associated with phenols content elevation, thereby influencing hypolipidemic or other bioactivities. A narrow, weak peak around 2930 cm^−1^ represented C–H stretching vibrations [42]. The peak at 1617 cm^−1^ represented the N–H variable angular vibration peak of the amino group, inferring that the cellulose had conjugated proteins [43]. The group of bands extending from 1485 to 1350 cm^−1^ were attributed to the presence of C-H within the methoxyl groups in lignin and hemicellulose. Meanwhile, their peak intensity for SE-SDF was higher than that for SDF, revealing a reduction in lignin and hemicellulose contents [44]. The hydrogen bond intensity (HBI) is used to reflect the ratio of the intensity of the peaks at 3405 cm^−1^ and 1402 cm^−1^. It is correlated with the crystal region and the amount of bound water [45]. The HBI showed a higher value of SE-SDF (0.33) and a lower value for the untreated materials (0.30), indicating an ordered cellulose structure after SE. The peak around 1260 cm^−1^ was related to the C=O ester linkages of the acetyl groups in hemicelluloses. The peak at 1261 cm^−1^ significantly moved forward, and its intensity decreased after the steam explosion, indicating the content and structural morphology changes in the hemicellulose [46]. A specific band at 1100–1000 cm^−1^ was the typical absorption of xylan. The prominent absorption at 1045 cm^−1^ corresponded to the C–O and C–C stretching or C–OH bending in hemicellulose [47]. With SE treatment, the absorption peak strength increased, which indicated that the breaking effect of the long chain of the hemicellulose leads to the exposure of the xylan structure. This is consistent with the increase in xylose, resulting in an improved lipid metabolism effect of SE-SDF. The peaks in the range of 350 to 600 cm^−1^ corresponded to the skeletal modes of pyranose rings [13]. The above results indicate that SE treatment breaks the long chain of the cellulose and hemicellulose, leading to the conversion of IDF to SDF with a relative increase in xylan.

### 3.3. Scanning Electron Microscopy

**Figure 4:**
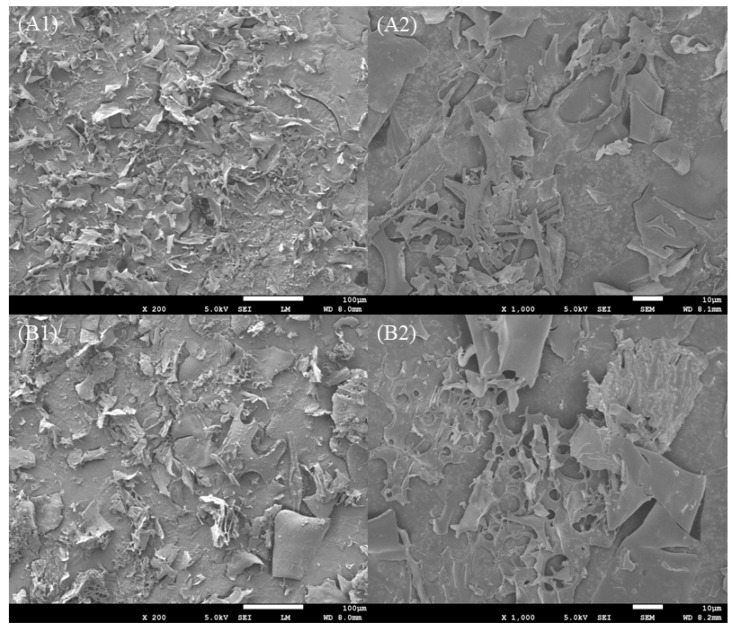
Scanning electron micrographs of SDF and SE-SDF. (**A**) SEM micrographs of SDF; (**B**) SEM micrographs of SE-SDF; (**1**) 200×; the size of the bar is 100 µm; (**2**) 1000×. The size of the bar is 10 µm.

Microstructures of *F. velutipes* SDF after SE modification are shown in Figure 4. The surface of SDF was lamellar and had a relatively smooth, scaly structure. After SE processing, an apparent variation in the SDF structure was observed. The surface began to lose its luster and tended to roughen, developing a porous, honeycombed structure. This change resulted from the explosion process of SE, in which the superheated steam in the sample vaporizes rapidly, and the volume expands sharply to generate huge shear forces, breaking fibers, generating pores, and finally destroying the originally tight structure [48]. These changes may improve the physicochemical properties and facilitate the conversion of IDF to SDF, thereby increasing the SDF yield and its functional properties [49]. Zhai et al. [50] also reported that SE could change the surface structure of *Rosa roxburghii* pomace SDF, and the surface progressed from smooth to rough and porous. Therefore, the SE treatment increased the surface area and porosity of the fiber, which could enhance its ability to bind cholesterol and bile acids in the gastrointestinal tract and their excretion, resulting in reducing their reabsorption, which might improve the hypolipidemic effect of SE-SDF [51,52].

### 3.4. Effect of SDF and SE-SDF on HepG2 Cell Viability and TG Content

**Figure 5:**
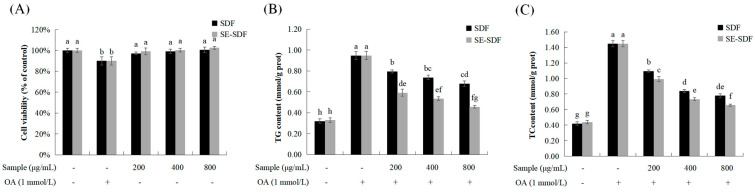
Effects of SDF and SE-SDF 0n oleic acid (OA)-induced obesity HepG2 cells: (**A**) influence of SDF and SE-SDF on cell viability; (**B**) impact of the co-treatment with the SDF, SE-SDF, and OA on the TG content; (**C**) influence of the co-treatment with SDF, SE-SDF, and OA on the TC content. Different letters indicate significant differences (*p* < 0.05). The minus and plus signs indicate whether sample and OA is added or not.

The MTT assay can assess the activity and proliferative capacity of cells. Measuring the amount of purple formazan formed through MTT reduction can indirectly reflect the metabolic activity and proliferative state of the cell [53]. In order to evaluate possible cytotoxicity, the HepG2 cells were treated with different concentrations (0, 200, 400, and 800 μg/mL) of SDF or SE-SDF for 24 h. The MTT color-reduction assay showed no significant toxicity for SDF or SE-SDF in cell viability over the entire tested concentration range of 0 to 800 μg/mL (Figure 5A). Therefore, these dosages were used for the SDF and SE-SDF treatments in the following experiments.

As an omega-9 monounsaturated fatty acid, oleic acid (OA) exists in all animal and plant oils in the form of glyceride, and it is the most ingested fatty acid in daily diets [54]. Lipid accumulation caused by OA induction in HepG2 cells is a commonly used in vitro model in the study of steatosis [55]. In our study, the TG and TC content was significantly increased (*p* < 0.05) through OA induction (Figure 5B,C), while SDF and SE-SDF treatment suppressed OA-induced increases in the content of TG and TC, with the dose of 800 μg/mL being the most effective (suppressed by 28.41% and 51.79% and 46.23% and 54.63%, respectively, for TG and TC content), indicating that the suppressive effect of SE-SDF was greater than that of SDF. In recent years, the hyperlipidemia reduction activities of active ingredients extracted from other plants have been investigated. The polysaccharide extracted from mulberry, named PFM-3, reduced lipid accumulation and TG by HepG2 cells. With 800 μg/mL PFM-3 treatment, TG accumulation induced by OA was significantly lowered by about 38% [56]. Ethanol extracts from *Potentilla anserina* L. attenuated OA-induced steatosis in HepG2 cells; ethanol extracts at 2.5 mg/mL reduced the lipid accumulation stimulated by the OA, along with the TG and TC contents by 40.14% and 33.94%, respectively [57]. Therefore, SDF and SE-SDF showed potential hyperlipidemia reduction activity. Furthermore, the effect of SE-SDF was better. This may be because SE-SDF has higher phenols, protein, and xylose content. Some recent studies have suggested that phenols and dietary fiber are promising candidates for prebiotics [58]. Furthermore, the rough and porous surface of SE-SDF could possibly enhance the ability to bind cholesterol and inhibit the absorption of cholesterol by the intestine, which might result in improving its hypolipidemic effect [52].

### 3.5. Effect of SE-SDF on Body Weight, Organ Weight, and Blood Lipid Levels of Mice

**Figure 6:**
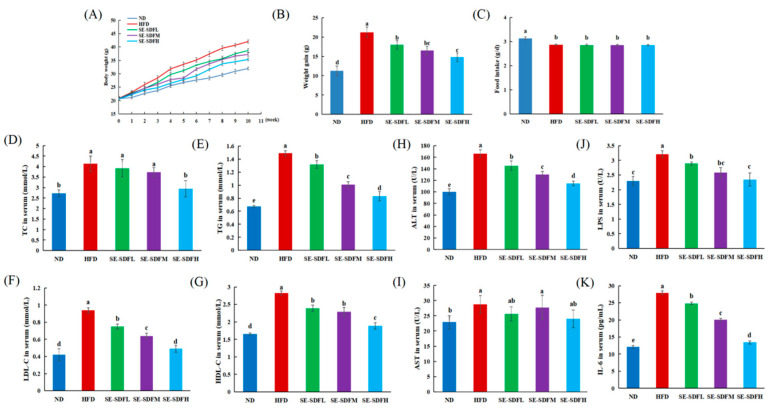
Effects of the soluble dietary fiber with steam explosion pretreatment (SE-SDF) in HFD-induced obese mice: (**A**) body weight; (**B**) weight gain; (**C**) food intake; (**D**–**G**) serum lipid levels of TC, TG, LDL-C, and HDL-C; (**H**,**I**) serum levels of ALT and AST; and (**J**,**K**) serum levels of LPS and IL-6. Different letters indicate significant differences (*p* < 0.05). (SE-SDFL: HFD + 50 mg/kg/d SE-SDF; SE-SDFM: HFD + 100 mg/kg/d SE-SDF; SE-SDFH: HFD + 200 mg/kg/d SE-SDF).

As demonstrated in Figure 6A,B, after 10 weeks, the body weight of the high-fat diet (HFD)-fed mice increased significantly in comparison with natural diet (ND)-fed mice (*p* < 0.05). Different doses of SE-SDF intervention suppressed the body weight gain in all treatment groups relative to the HFD group (*p* < 0.05). A previous study reported that the intervention of polysaccharide at 50, 100, and 200 mg/kg remarkably inhibited both the weight gain in mice and the rate of weight gain [59]. No significant difference in food intake was observed among the HFD-fed groups, and mice in the ND group fed the normal diet consumed the most (Figure 6C), indicating that the impact of SE-SDF in reducing the weight gain of obese mice was not due to reducing food consumption.

Four lipid indicators (TG, TC, LDL-C, and HDL-C) in the blood were determined to elucidate the influence of SE-SDF on lipid levels in mouse blood (Figure 6D–G). The HFD group showed higher blood lipid levels in comparison with those in other treatments (*p* < 0.05). Nevertheless, daily interventions of SE-SDF reduced blood lipid levels of TC, TG, and LDL-C in comparison with the HFD group. Notably, for high doses of SE-SDF, the levels of TC and LDL-C differed significantly from the values for the HFD group (*p* < 0.05) in assessing the inhibitory effect of polysaccharides on blood lipid levels. Zhao et al. [60] found that blood levels of TC, TG, and LDL-C were largely decreased by pretreatment with 400 mg/kg of polysaccharide from *F. velutipes*, consistent with our results. Ji et al. [61] proposed that pear pomace SDF with a 1–5 g/kg intervention can effectively improve metabolic disorders in HFD-fed mice, mainly by reducing the levels of TC, TG, and LDL-C in the blood, increasing the level of HDL-C. Thus, the above data indicate that the inhibitory effect of SE-SDF on blood lipid indicator levels might improve hyperlipidemia in obese mice.

The ALT and AST in serum are significant indicators and are used to evaluate liver injury. The content of ALT in the HFD group was higher than that in the control group, while the value in the SE-SDF group was close to the control (Figure 6H). Further, ALT content in the SE-SDF group was lower than in the HFD group (*p* < 0.05), with values of 31.11% (SE-SDFH group), 21.92% (SE-SDFM group), and 11.48% (SE-SDFL group), respectively. These results suggested that SE-SDF could reduce liver damage and protect liver health.

Moreover, HFD increased serum lipopolysaccharide (LPS) and interleukin 6 (IL-6) levels, and SE-SDF treatment led to their downregulation (Figure 6J,K). Nguepi Tsopmejio et al. [62] reported that *F. velutipes* extracts in mice showed an anti-inflammatory action, indicated by the inhibition of Toll-like receptor 4 (TLR4)/nuclear factor-κB (NF-κB) levels, and the expression of related signaling pathways, thereby attenuating the expression of the genes and proteins related to lipid metabolism and lowering lipid synthesis.

### 3.6. Effect of SE-SDF on Liver Biochemical Parameters and Oxidative Stress in HFD-Fed Mice

**Figure 7:**
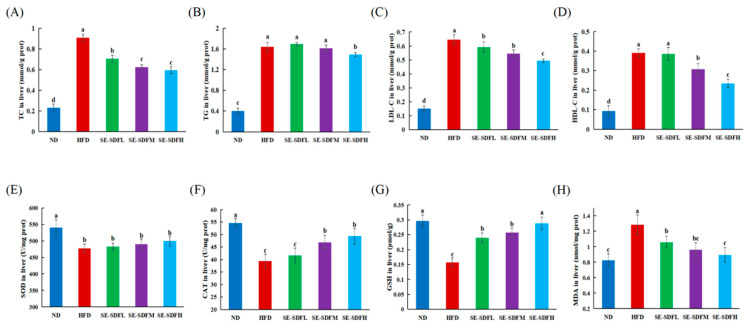
Effects of the soluble dietary fiber with steam explosion pretreatment (SE-SDF) on liver parameters and oxidative stress in HFD-induced obese mice: (**A**–**D**) Liver levels of TC, TG, LDL-C, and HDL-C; (**E**–**H**) liver levels of SOD, CAT, GSH, and MDA. Different letters indicate significant differences (*p* < 0.05). (SE-SDFL: HFD + 50 mg/kg/d SE-SDF; SE-SDFM: HFD + 100 mg/kg/d SE-SDF; SE-SDFH: HFD + 200 mg/kg/d SE-SDF).

Long-term exposure of mice to a high-fat diet will result in hyperlipemia in the serum and liver lipid metabolism disorder, including lipid deposition and high levels of TC and TG, which causes liver injury. Studies have revealed that excessive intakes of triglycerides and cholesterol by mice cause cytotoxicity and damage to hepatocyte function, resulting in decreased TG transport ability in hepatocytes and further tissue damage [63,64]. To assess the effects of SE-SDF on the liver, we determined four biochemical parameters of liver lipids. The HFD intervention markedly increased liver TC, TG, and LDL-C levels in comparison with ND (Figure 7A–D). However, the TC, TG, and LDL-C levels were dose-dependently downregulated by SE-SDF administration; in particular, high-dose SE-SDF reduced TC and LDL-C by 34% and 24%. Caz et al. [65] also found that SDF from *Pleurotus ostreatus* could reduce liver TG levels in mice. In addition, Luo et al. [66] reported that the bamboo shoot shell fibers alleviated the disorderly situation of lipid metabolism of hyperlipidemia mice. With total DF intervention, the TC, TG, and LDL-C decreased by 31.53%, 21.35%, and 31.53%, respectively. Consequently, the above data imply that the inhibitory effect of SE-SDF on lipid factors might attenuate the degree of hyperlipidemia.

Obesity results in chronic low-grade inflammation, accompanied by a variety of metabolic disorders [67]. According to reports, the abnormal or excessive accumulation of adipose tissue might be related to an increase in pro-oxidative responses [68]. As shown in Figure 7E–H, the hepatic activities of SOD and CAT and the GSH level were reduced in the HFD group, but the levels of MDA were significantly higher in the HFD group compared to the ND group. The SE-SDF intervention significantly increased the activity of antioxidant enzymes such as CAT and GSH and decreased oxidative mediator (MDA) levels. In particular, for the high-dose SE-SDF groups, the levels of GSH and MDA did not differ from those of the ND group (*p* > 0.05). Several researchers have linked the hypolipidemic activity of polysaccharides with antioxidant action via regulating the Nrf2-keap1 or AMPK signaling pathway and suppressing the production and reaction of the oxidative mediators and oxidative-related enzymes (e.g., ROS, MDA, GSH, SOD, and CAT) to alleviate lipid accumulation and lipid oxidation [35,69]. The water-soluble dietary fiber extracted from walnut meal effectively reversed the lipid metabolism disorder, enhanced antioxidant capability, and alleviated inflammation to prevent metabolic diseases caused by HFD in mice [70]. Meng et al. [71] found that burdock fructooligosaccharide improved the antioxidant enzyme activities and alleviated hepatocyte damage, thereby ameliorating hypercholesterolemia. Therefore, our study revealed that SE-SDF could improve the oxidative stress in liver tissue caused by an HFD, which promoted proper lipid metabolism, thus reducing the accumulation of lipids in the liver.

### 3.7. Effect of SE-SDF on Liver Tissue Morphology in Mice

**Figure 8:**
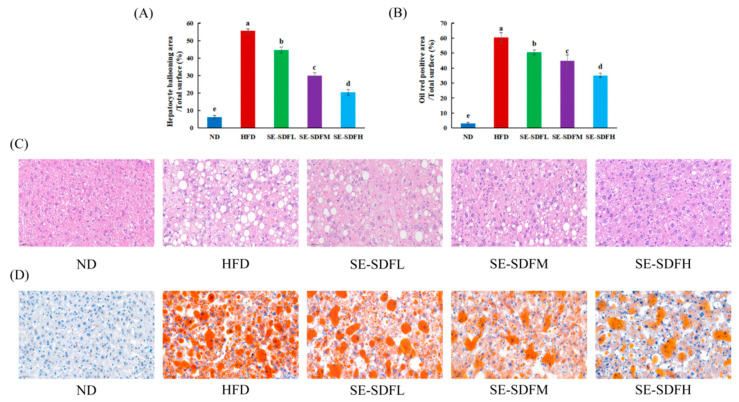
Effects of soluble dietary fiber with steam explosion pretreatment (SE-SDF) on hepatic function and histopathology in HFD-induced obese mice: (**A**) The quantification of the hepatocyte vacuolation area; (**B**) the quantification of the Oil Red positive area; (**C**) HE staining; (**D**) Oil Red staining. Different letters indicate significant differences (*p* < 0.05).(SE-SDFL: HFD + 50 mg/kg/d SE-SDF; SE-SDFM: HFD + 100 mg/kg/d SE-SDF; SE-SDFH: HFD + 200 mg/kg/d SE-SDF).

Microscopic observations of liver histological sections stained with hematoxylin–eosin (HE) (Figure 8C) showed that the nucleus and cytoplasm of the liver tissue were stained blue and red, respectively. The percentage of hepatocyte vacuolation area in liver tissue cells is shown in Figure 8A. The results showed a percentage of vacuolated liver histiocytes was elevated in HFD-fed mice compared to the ND group. The SE-SDF intervention reduced the number of vacuoles and inflammatory infiltration in cells. Meanwhile, the Oil Red O staining of liver tissue demonstrated significantly abnormal lipid accumulation in the liver in the HFD group in comparison with the ND group. The SE-SDF groups reversed this phenomenon, and both the number and area of lipid droplets declined after different doses of SE-SDF intervention (Figure 8B,D). This demonstrated that SE-SDF effectively alleviated lipid accumulation and liver damage in HFD-fed mice.

In our study, SE-SDF intervention improved lipid profiles and reduced liver damage in obese mice, possibly by inhibiting key genes involved in triglyceride synthesis and lipid metabolism, thereby helping to prevent obesity. Tang et al. [72] reported that β-glucans exhibited a dose-dependent hypolipidemic effect on HFD-induced mice; the β-glucans suppressed the body weight, organ weight, serum and liver levels (e.g., TG, TC, LDL-C), and the lipid accumulation of adipose and liver tissues, while lowering the expression of PPARγ, C/EBPα, SREBP-1c, FAS, SCD1, and LPL and lifting the expression of CPT-1 and HSL in the AMPK signaling pathways. Li et al. [73] explored whether *Grifola frondosa* polysaccharide could upregulate the expression of AMPK-α, PPARα, GCK, and CYP7A1 and downregulate the expression of SREBP-1c, FAS, and ACC, thereby exerting a hypolipidemic effect. Therefore, it is reasonable to hypothesize that SE-SDF may regulate lipid metabolism through these protein pathways due to its improved structural features and desirable effects on lipid levels. In future experiments, we plan to further explore the hypolipidemic mechanism of SE-SDF through these pathways, particularly AMPK, PPAR, and SREBPs (e.g., the proteins AMPK-α, PPARα, and SREBP-1c). Therefore, SE-SDF intervention can ameliorate hyperlipidemia in obese mice by reducing blood lipids and liver damage and improving the antioxidant capacity of the liver, but these results are insufficient to guide clinical recommendations owing to the limitations of our animal experimental design. Moreover, the detailed function mechanism requires further exploration.

Overall, the SE process is a useful method by which to elevate SDF yield and enhance functional activities. However, the techno-economic feasibility of extracting SDF from edible fungi process waste through the SE process at the industrial scale should be further evaluated from the perspectives of equipment scale-up, energy consumption, combined processing, and acquiring valuable products. According to the literature, SE equipment has been reported to be scaled up from the laboratory scale to the industrial scale (up to 50 m^3^) to satisfy the requirements of vast biomass material refining [74,75]. In order to improve the process economy, an appropriate hydration pretreatment, the regulation strategy for the SE parameters, and a mixture with other biomass materials should be considered. This hydrothermal modification technology not only effectively appreciates the undervalued fungi resources but also reduces the ecological damage and environmental pollution caused by edible fungi process wastage. In addition to SDF, more active ingredients such as ergosterol, chitosan, and dextran can be combined to prepare functional foods, cosmetics, and drugs in order to fully improve the added value of waste.

## 4. Conclusions

We provided a novel SDF, SE-SDF, with a high xylose content that was extracted from *F. velutipes* root via SE pretreatment. Compared to SDF, the carbohydrate and protein contents of SE-SDF were increased to 87.71% and 3.51%, respectively, with the former being composed of Rha, Fuc, Xyl, Man, Gal, Glc, and Ara, indicating that SE-SDF is a heteroglycan. SE-SDF reduced lipid accumulation in OA-induced HepG2 cells. The SE-SDF had no toxicity at concentrations of 200–800 μg/mL and suppressed TG content in a dose-dependent manner. Furthermore, it has been shown to possess an alleviative effect in obese HFD mice by reducing blood lipids and liver damage, improving the antioxidant capacity of the liver. This work provides an effective method for the processing of fungi waste, the SE-SDF can be applied in the research and development of healthcare products. In future studies, the structural characteristics and the anti-obesity and gut microbiota regulation mechanisms of SE-SDF will be explored in depth. Studies of SE-SDF in hyperlipidemic or obese people in effective doses, focusing on bioavailability and the regulation mechanism, will be required.

## Figures and Tables

**Table 1:** Chemical content, monosaccharide composition, and molecular weight of SDF and SE-SDF.

Sample	Total Sugar (%)	Phenol (%)	Protein (%)	Monosaccharide Composition (%)							Molecular Weight (kDa)
				Rha	Fuc	Xyl	Man	Gal	Glu	Ara	
SDF	82.68 ± 0.27	0.69 ± 0.04	2.16 ± 0.09	3.66 ± 0.01	5.60 ± 0.03	9.25 ± 0.03	8.81 ± 0.01	13.25 ± 0.02	56.74 ± 0.08	2.70 ± 0.01	312.5
SE-SDF	87.71 ± 0.42	0.84 ± 0.03	3.51 ± 0.05	2.06 ± 0.01	2.92 ± 0.01	14.66 ± 0.02	17.91 ± 0.03	7.82 ± 0.01	52.54 ± 0.05	2.10 ± 0.01	122.7

## Data Availability

The original contributions presented in the study are included in the article/Supplementary Materials, further inquiries can be directed to the corresponding authors.

## Supplementary Materials

The following supporting information can be downloaded at: https://www.mdpi.com/article/10.3390/foods13223621/s1, Figure S1. Single factor test of steam explosion on the SDF yield in *F. velutipes* root; Table S1: Orthogonal experiment results; Table S2: Compositions of experimental diets.
