# Supplementary material for: Enhancing the Hypolipidemic and Functional Properties of Flammulina velutipes Root Dietary Fiber via Steam Explosion
Source: Foods. 2024 Nov 13;13(22):3621. doi: 10.3390/foods13223621 (PMC11593700; doi:10.3390/foods13223621)
Supplement: Supplementary file 1 [file foods-13-03621-s001.zip › foods-3260765-supplementary.pdf]

Enhancing the Hypolipidemic and Functional Properties of *Flammulina velutipes* Root Dietary Fiber via Steam Explosion

Supplementary Figure S1

Supplementary Tables S1 and S2

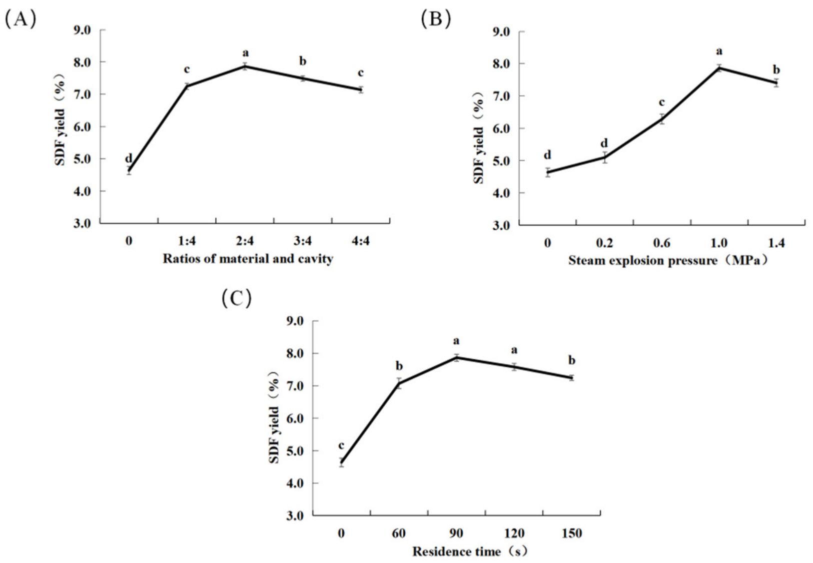

Supplemental Figure S1. Single factor test of steam explosion on the SDF yield in *F. velutipes* root, (A) Ratios of material and cavity; (B) Steam explosion pressure; (C) Residence time.

Supplemental Table S1. Orthogonal experiment results

Number	Factors			SDF yield （%）
	Ratios of material and cavity	Residence time (s)	SE pressure (MPa)	
1	1(3:8)	1（75）	1（0.8）	7.91±0.08
2	1	2（90）	3（1.2）	8.05±0.13
3	1	3（105）	2（1.0）	8.12±0.16
4	2(4:8）	1	3	7.93±0.07
5	2	2	2	8.03±0.11
6	2	3	1	7.96±0.09
7	3(5:8)	1	2	8.10±0.12
8	3	2	1	7.95±0.08
9	3	3	3	8.17±0.14
k_1	8.03	7.98	7.94	
k_2	8.00	8.01	8.08	
k_3	8.07	8.08	8.05	
R	0.07	0.10	0.14	

Supplemental Table S2. Compositions of experimental diets

Ingredients (gm)	Normal diet	High-fat diet
Casein, 80 Mesh	200	200
L-Cystine	3	3
Com Starch	506.2	0
Maltodextrin 10	125	125
Sucrose	68.8	68.8
Cellulose, BW200	50	50
Soybean Oil	25	25
Lard	20	245
Mineral Mix S10026	10	10
DiCalcium Phosphate	13	13
Calcium Carbonate	5.5	5.5
Potassium Citrate, 1 H_20	16.5	16.5
Vitamin Mix V10001Choline Bitartrate	10	10
Choline Bitartrate	2	2
